# Approach Based on the Ordered Fuzzy Decision Making System Dedicated to Supplier Evaluation in Supply Chain Management

**DOI:** 10.3390/e26100860

**Published:** 2024-10-12

**Authors:** Katarzyna Rudnik, Anna Chwastyk, Iwona Pisz

**Affiliations:** 1 Faculty of Production Engineering and Logistics, Opole University of Technology, 45-758 Opole, Poland; 2 Centre for Education and Applications of Mathematics, Opole University of Technology, 45-758 Opole, Poland; a.chwastyk@po.edu.pl; 3 Faculty of Economics, University of Opole, 45-040 Opole, Poland; ipisz@uni.opole.pl

**Keywords:** multi-criteria decision making, supplier, supplier evaluation, supply chain, fuzzy logic, ordered fuzzy number, fuzzy system, ordered fuzzy decision making system, risk, ESG, entropy

## Abstract

The selection of suppliers represents a pivotal aspect of supply chain management and has a considerable impact on the success and competitiveness of the organization in question. The selection of a suitable supplier is a multi-criteria decision making (MCDM) problem based on a number of qualitative, quantitative, and even conflicting criteria. The aim of this paper is to propose a novel MCDM approach dedicated to the supplier evaluation problem using an ordered fuzzy decision making system. This study uses a fuzzy inference system based on IF–THEN rules with ordered fuzzy numbers (OFNs). The approach employs the concept of OFNs to account for potential uncertainty and subjectivity in the decision making process, and it also takes into account the trends of changes in assessment values and entropy in the final supplier evaluation. This paper’s principal contribution is the development of a knowledge base and the demonstration of its application in an ordered fuzzy expert system for multi-criteria supplier evaluation in a dynamic and uncertain environment. The proposed system takes into account the dynamic changes in the value of assessment parameters in the overall supplier assessment, allowing for the differentiation of suppliers based on current and historical data. The utilization of OFNs in a fuzzy model then allows for a reduction in the complexity of the knowledge base in comparison to a classical fuzzy system and makes it more accessible to users, as it requires only basic arithmetic operations in the inference process. This paper presents a comprehensive framework for the assessment of suppliers against a range of criteria, including local hiring, completeness, and defect factors. Furthermore, the potential to integrate sustainability and ESG (environmental, social, and corporate governance) criteria in the assessment process adds value to the decision making framework by adapting to current trends in supply chain management.

## 1. Introduction

In the contemporary competitive environment, organizations are increasingly reliant on strategic relationships with their suppliers to develop value-added systems that afford them a competitive advantage in the marketplace. The smooth functioning of a business is contingent upon a robust supply chain, which in turn enables the delivery of value to customers [1,2]. Supplier selection is of great consequence in this process. Choosing of an inappropriate supplier can result in a series of adverse consequences, including a decline in product quality, delayed deliveries, and elevated costs. The implementation of supplier evaluation methods provides a structured framework to mitigate these risks and identify the most suitable suppliers for a company’s specific requirements. Supplier selection is a pivotal aspect of supply chain management, with the outcome having a considerable impact on the success and competitiveness of the organization. It is of paramount importance for any business to select the appropriate suppliers to ensure efficient operations, cost optimization, and overall success [3,4].

Supplier evaluation methods provide a systematic approach to the assessment of potential and existing suppliers against predefined criteria. The evaluation of suppliers represents a pivotal aspect of supply chain management whereby organizations can ascertain the suitability of suppliers in terms of their ability to consistently deliver quality products and services [5,6,7,8]. This review synthesizes the key literature on the subject of supplier evaluation to highlight the methodologies, criteria, and frameworks that are currently in use in a variety of different industries.

Conversely, the maintenance of sustainability represents a significant challenge for contemporary businesses and supply chains [9,10,11]. Environmental, social, and governance (ESG) practices represent a framework for the management of sustainability, ethical practices, and conscious consumerism that is gaining considerable traction in the business world. In light of the growing negative impacts of organizations’ actions, it is imperative that businesses measure and make public their environmental and social performance [12,13]. In addition to financial metrics, sustainability metrics include factors, such as natural resource conservation, emission levels, environmental initiatives, occupational health and safety, community relations, stakeholder engagement, and the economic impact. It is important to note that investors, customers, and the public are increasingly paying attention to companies’ actions related to environmental protection, human resource management, ethical conduct, and transparent governance [14].

The integration of ESG principles into supply chains entails the reduction of adverse environmental impacts, the minimization of carbon footprints, the promotion of fair working conditions, and the development of a sustainable economy with the selection of appropriate suppliers of products and services, which is of significant importance from a value chain perspective. The discussion of environmental, social, and corporate governance criteria within the concept of corporate sustainability has gained considerable momentum in recent years. This is driven by evolving social expectations for new production and consumption models [13,14,15]. Until the mid-1990s, the success of companies was primarily contingent upon their ability to meet the needs of a single stakeholder. Subsequently, other stakeholders exerted pressure on companies, which resulted in the incorporation of ESG management criteria into the strategic management process [16]. It can be asserted that there is a growing focus on the sustainability performance of all parties within the supply chain. The evaluation of supplier sustainability is becoming an increasingly significant aspect [17]. The assessment of suppliers in accordance with sustainable criteria constitutes an essential element of the process of transforming corporate sustainability and ESG (environmental, social, and governance) practices, and it exerts a significant influence on the behavior of companies in the selection of suppliers [18]. This is particularly the case for manufacturing companies, where the sustainability of the products supplied directly determines the sustainable performance of the final goods produced [19].

Numerous methods have been employed to evaluate suppliers, each with a unique set of criteria tailored to specific industry needs and strategic goals. The most commonly used methods include multi-criteria decision making (MCDM) approaches, such as the Analytic Hierarchy Process (AHP) and the Technique for Order Preference by Similarity to Ideal Solution (TOPSIS), as well as mathematical programming techniques, like Data Envelopment Analysis (DEA) and mixed-integer linear programming [7]. These models enable companies to evaluate suppliers based on critical criteria, including quality, cost, delivery performance, flexibility, and sustainability, with sustainability and risk factors gaining increasing prominence in recent years [5]. Hybrid approaches, which combine multiple techniques, have also been proposed to address complex supply chain environments and incorporate both qualitative and quantitative factors, reflecting the evolving requirements of supplier evaluation frameworks [8]. This body of literature underscores the need to apply comprehensive evaluation models to align supplier capabilities with organizational objectives while also adapting to dynamic industry standards.

One of the principal challenges inherent in the evaluation of suppliers is the necessity to address the inherently dynamic nature of the business environment. This research presents an approach to supplier evaluation and selection using an ordered fuzzy decision making system. This approach offers a more robust framework for supplier selection in dynamic business environments. OFNs provide supplementary information in the form of the orientation of the fuzzy numbers (positive or negative). Depending on the context, this information can be interpreted in different ways. For example, it can be used to represent the trend and dynamics of change [20,21,22], it can serve as a type of criterion (cost, benefits) [23], or it can reflect the “at most” and “at least” relationship to fuzzy numbers [24].

In recent years, the OFN concept has undergone significant development, with a growing number of applications across various practical fields. These include ranking evaluated negotiation offers [24,25], monitoring of crisis control centers [26], critical path analysis [21], improvement project evaluations [27], data volatility forecasting [28,29], solving vehicle routing and scheduling problems [30], modeling of limit order book data [31], and analysis of carbon price volatility [32].

A review of the literature reveals that the majority of studies concentrate on the development or application of particular methods for arithmetic calculations or analysis utilizing OFNs. Nevertheless, there is a paucity of literature examining the issue of inference from a knowledge base in the form of rules with OFNs, which are referred to as ordered fuzzy rules. In a fuzzy control application, rules from OFNs were limited to set-value tables without the use of an inference procedure [33]. An alternative approach to fuzzy inference without a rule base was employed by the author of [34], where inference was based on a mathematical rule. In [35], a proposal for the creation of if–then models with OFNs based on incremental quantitative data was presented.

In order to address this research gap, we have modified the concept of the inference method proposed by Prokopowicz [36], who is the author of the only known inference mechanism for ordered fuzzy rules. This concept was outlined by the authors in the IEEE FUZZ 2023 post-conference monograph [37]. This article builds upon this concept. The article’s principal contribution is the development of a knowledge base and the demonstration of its application in an ordered fuzzy expert system for multi-criteria supplier evaluation in a dynamic and uncertain environment. The proposed system considers the dynamic changes in the value of assessment parameters in the overall supplier assessment, thus allowing for the differentiation of suppliers based on current and historical data. The utilization of OFNs in a fuzzy model then permits a reduction in the complexity of the knowledge base in comparison to a classical fuzzy system. The general inference procedure [36] was proposed for a complex system and modified to make it more sensitive to the direction of OFNs in the rules. The presented research represents a significant advancement in the development of the OFN concept, paving the way for its practical application in managerial contexts.

The paper is structured as follows. Section 2 presents a review of the literature and provides an overview of supplier evaluation and selection, including the use of fuzzy logic. Section 3 outlines a proposed system for the evaluation of suppliers that employs a fuzzy inference system with OFNs. Section 4 presents an empirical illustration of the implementation of this system. Section 5 elucidates the results and offers a discussion to demonstrate the efficacy and applicability of the proposed approach in the context of supplier evaluation. Finally, Section 6 presents the conclusions.

## 2. Related Work

### 2.1. Literature Review of Supplier Evaluation and Selection

Supplier evaluation is a time-consuming activity and a critical step in ensuring the quality, safety, and sustainability of the business and the supply chain. Selecting the right suppliers is critical for any organization to ensure efficient operations, cost optimization, and overall success. Supplier evaluation methods provide a systematic approach to evaluating potential and existing suppliers against pre-defined criteria.

In light of the findings of the literature review, it is imperative to emphasize the significance of clearly delineated objectives in generating value for the evaluation company [38]. The evaluation criteria employed in the selection of suppliers are of paramount importance [39]. Over the past two decades, the study of methods and criteria for supplier selection has been a central topic in literature reviews. Subsequently, uncertainty management, environmental management, and sustainability have emerged as relevant topics in literature reviews from 2013 onwards [40]. One of the principal challenges in the evaluation of suppliers is the necessity of dealing with the dynamic nature of business environments. Handfield et al. [41] discuss the requirement for continuous monitoring and re-evaluation of suppliers in order to adapt to changing market conditions and organizational needs. The advent of big data and advanced analytics offers new opportunities for the evaluation of suppliers. Dubey et al. [42] explore how big data analytics can enhance decision making by providing deeper insights into supplier performance and market trends.

A review of the literature on the evaluation and selection of suppliers reveals a recent focus on uncertainty management. For example, in their respective studies, refs.[43,44,45,46,47] investigate supplier selection in non-deterministic environments, emphasizing the predominance of techniques based on fuzzy logic. Authors in [3,4,48,49,50,51] present comprehensive literature reviews on the subject of green supplier selection. Similarly, authors in [52,53] offer detailed reviews on the topic of sustainable supplier selection. Zailani et al. [54] propose the development of more robust frameworks that balance economic, environmental, and social considerations. A number of models and methods have been associated with the successful evaluation and selection of suppliers. Research indicates that a variety of analytical approaches and methods have been developed for supplier selection. Table 1 provides a comprehensive overview of the methodologies discussed in the literature and their key applications in supplier evaluation and selection.

Several studies have integrated multiple MCDM methods to enhance supplier evaluation processes. The Analytic Hierarchy Process (AHP) and the Analytic Network Process (ANP) are widely recognized techniques used in multi-criteria decision making. Saaty [55] introduced AHP, which has been widely adopted for supplier evaluation. It involves decomposing the decision problem into a hierarchy of more easily comprehended sub-problems, each of which can be analyzed independently. The elements of the hierarchy are then evaluated using pairwise comparisons, and the results are used to derive priority scales. ANP, as an advanced version of AHP, extends its capabilities by accounting for interdependencies among decision elements, thus allowing for more complex scenarios. Together, AHP and ANP are considered essential tools in modern decision making frameworks, particularly in the context of supplier selection. Case studies across various industries demonstrate the practical application of these methods. For example, Büyüközkan and Çifçi [56] use fuzzy AHP and fuzzy TOPSIS to evaluate suppliers in the automotive industry, highlighting the effectiveness of integrated fuzzy MCDM approaches. DEMATEL is a modeling technique used to examine and understand the interrelationships and influence between complex evaluation criteria in a structured manner. It helps visualize and map the cause-and-effect relationships among various factors to better analyze their impact [43]. Linear Programming (LP) is a mathematical technique used to find the optimal solution for a given problem by maximizing or minimizing a specific objective function. It works by analyzing a set of linear equations and inequalities that define the constraints, thus ensuring that the optimal solution satisfies all of the specified requirements [57]. Another method like DEA has been applied in various studies to evaluate supplier performance while considering multiple criteria. DEA, developed by Charnes et al. [58], is a non-parametric method used to evaluate the efficiency of decision making units (DMUs), such as suppliers. It compares multiple input and output measures to assess the relative efficiency of suppliers. DEA has been applied in various studies to evaluate supplier performance while considering multiple criteria. Ho et al. [55,59] combine AHP and DEA to leverage the strengths of both methods, thus providing a more comprehensive evaluation framework.

It can be stated that there has been a discernible shift towards more sophisticated and integrated approaches to supplier selection over time, incorporating a range of criteria and advanced analytical techniques. Supplier criteria can be classified into two main categories: quantitative and qualitative attributes.

The selection of appropriate criteria is also contingent upon the specific purchasing situation. The primary criteria typically evaluated in supplier selection include the following [3,4,5,6]:

- quality,
- cost/price,
- delivery performance,
- flexibility,
- environmental aspects,
- sustainability,
- warranties and claim policies,
- geographical localization, etc.

In supplier selection, relevant criteria are those that have a significant impact on the operational success of the supply chain. The criteria include critical aspects of supply chain operations that can affect overall business success. The evaluation of suppliers depends on the evaluation of quality. As posited by Dickson [60], quality represents one of the most pivotal considerations for purchasing managers. The findings of studies conducted by Weber et al. [61] and Choy et al. [62] serve to reinforce the significance of quality, underscoring the necessity for meticulous quality control and assurance procedures. Cost is another critical factor that must be considered. Ellram [63] proposes that a total cost analysis, encompassing the purchase price, transaction costs, and logistics costs, offers a comprehensive perspective on the financial implications of supplier selection. A cost–benefit analysis is frequently employed for the purpose of comparing suppliers. The timely delivery of goods is of paramount importance for the maintenance of production schedules and the fulfilment of customer orders. The occurrence of late deliveries has the potential to disrupt operational processes and result in increased costs. Lai et al. [64] emphasize the significance of delivery reliability and flexibility as pivotal performance indicators. The term “flexibility” is used to describe a supplier’s ability to respond to changes in demand and adapt to new requirements. Lummus et al. [65] discuss the increasing necessity for supplier flexibility in dynamic market environments, emphasizing its role in competitive advantage. The technological capability of suppliers has an impact on their capacity to innovate and deliver advanced products. Prahinski and Benton [66] posit that the evaluation of suppliers’ technological capabilities is crucial for the establishment of long-term partnerships and the facilitation of collaborative innovation. The recent literature has increasingly focused on sustainable procurement practices. Carter and Rogers [67] explore the integration of environmental and social criteria into supplier evaluation, emphasizing the importance of corporate social responsibility (CSR) in supplier relationships. Luthra et al. [68] propose sustainability criteria, which refer to the supplier’s ability to maintain and promote social, economic, and environmental sustainability in their operations.

The literature on supplier evaluation and selection highlights a wide array of methods, ranging from traditional multi-criteria decision making (MCDM) techniques, such as AHP and DEA, to more advanced hybrid models. In recent years, there has been a notable shift towards incorporating sustainability, risk management, and big data analytics into the evaluation frameworks. The integration of these elements is crucial to adapt to the dynamic nature of modern supply chains and evolving business environments. Despite the extensive body of research, a gap remains in the development of robust frameworks that balance traditional performance criteria (quality, cost, delivery) with newer dimensions, such as environmental and social sustainability, especially in uncertain and complex decision making environments. While many studies focus on individual approaches, there is a lack of comprehensive frameworks that integrate these diverse criteria in a systematic manner, particularly for non-deterministic scenarios.

### 2.2. Fuzzy Logic in the Supplier Evaluation Method

Fuzzy logic, introduced by Zadeh [69], deals with reasoning that is approximate rather than fixed and exact. It has been applied in supplier evaluation to handle the uncertainty and subjectivity in the decision making process. Most studies use fuzzy logic or its modifications to evaluate suppliers in extensions of MCDM methods. This allows both quantitative and qualitative criteria to be included in the evaluation (e.g., [70]). In these approaches, fuzzy logic is often used to evaluate supplier performance based on linguistic terms (e.g., “good”, “excellent”), which works well in describing the vagueness associated with human evaluation. A range of fuzzy-based techniques are employed in multi-criteria evaluation approaches, including fuzzy AHP (Analytic Hierarchy Process), fuzzy TOPSIS (Technique for Order Preference by Similarity to an Ideal Solution) [71,72], fuzzy DEA (Data Envelopment Analysis), and other techniques, along with various combinations.

The integrated Fuzzy Analytical Hierarchy Process–Combined Compromise Solution (Fuzzy AHP-CoCoSo) and Fuzzy Stepwise Weight Assessment Ratio Analysis–Failure Mode and Effects Analysis (Fuzzy SWARA-FMEA) are employed for the assessment of suppliers based on green criteria and supply risks [70]. The final selection is determined by aggregating the overall score using Data Envelopment Analysis (DEA). The Pythagorean fuzzy DEA methodology has been proposed by Shao et al. [73]. Furthermore, the authors proposed a novel fuzzy MA-VIKOR sub-model based on heterogeneous data, which can evaluate suppliers from two perspectives, group utility and individual regret, while avoiding the potential problem of equal ranking.

In the study [74], the authors employed interval type-2 fuzzy sets to quantify the inputs provided by decision makers and proposed an extended super-efficiency DEA model. This model encompasses both desirable and undesirable inputs and outputs, allowing for the evaluation of suppliers. The Fuzzy ANP (Analytic Network Process) method is an extension of the traditional AHP method that takes into account interactions and feedback between criteria. Thanks to fuzzy logic, the process can cope with imprecise human judgements, making it more effective in supplier evaluation [75]. A combination of QFD (Quality Function Deployment) and fuzzy TOPSIS techniques is employed for the evaluation of suppliers, taking into account both quantitative and qualitative criteria. This approach enables the assessment of suppliers in terms of product alignment, thereby facilitating a more precise alignment of suppliers with the needs of the company [76]. Moreover, Ashish et al. [77] demonstrated that gray relational analysis with fuzzy logic can be effectively employed for the resolution of multi-criteria supplier selection issues.

An alternative fuzzy approach to supplier evaluation is to construct a fuzzy system based on a knowledge base, which is most often created from experts’ knowledge [78,79] or automatically created based on learning from historical data. These systems may be either single-inference elements or multi-stage hierarchical combinations of assessments [80,81]. In the study [82], the authors also propose a system with a fuzzy neural network, which can be developed through continuous learning and iterative modification. The authors of [83] propose that the FIS method is superior to the factor weighting method, as it allows for more effective management of the non-linear behavior and uncertainty inherent to the supplier evaluation process.

In conclusion, the literature review demonstrates that the combination of fuzzy logic with various multi-criteria decision making methods provides a valuable tool for supplier selection. This enables companies to make informed decisions in the face of uncertainty and imprecise data. These methods assist in the reduction of bias associated with the attribution of unfavorable numerical results to subjective assessments and facilitate the modeling of intricate relationships between criteria and supplier performance. It is a limitation of the current approaches that they analyze supplier evaluation criteria in a static manner without considering the dynamics and changes that occur over time. Areas for further research include the integration of various fuzzy methods with other artificial intelligence (AI) and machine learning (ML) techniques with the aim of better dealing with dynamic business environments. Additionally, greater integration of fuzzy methods with big data technologies could improve the accuracy of supplier assessments.

## 3. Proposed System for Evaluating Suppliers Using a Fuzzy Inference System with OFNs

**Figure 1:**
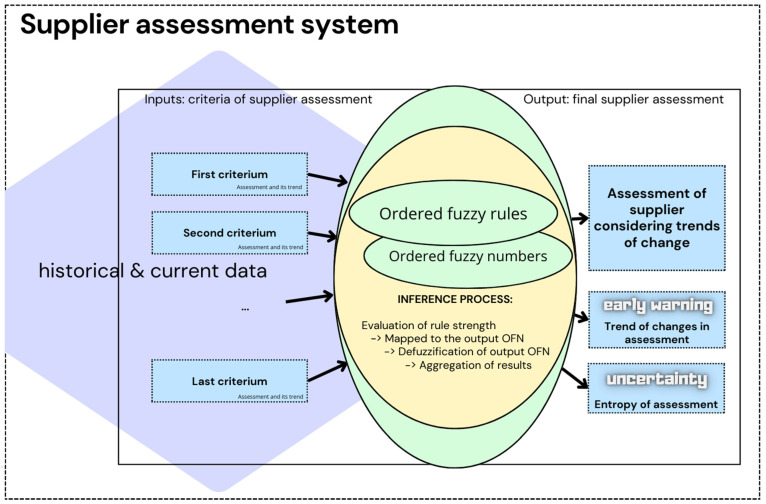
Supplier assessment system.

The objective of this section is to propose a transparent supplier evaluation system to determine the value of a scoring measure that determines the quality of suppliers from the perspective of current cooperation activities and based on their history. The evaluation system comprises a Fuzzy Inference System (FIS), which formalizes the human reasoning process using fuzzy logic based on rules written using ordered fuzzy numbers. The OFNs are employed to ascertain the descriptive value (linguistic values) of supplier evaluations in accordance with the specified criteria, along with the trend of changes in these evaluations, determined on the basis of data collected from periods preceding the evaluation. The structure of a system for evaluating suppliers using a fuzzy inference system with OFNs is illustrated in Figure 1.

### 3.1. Concept of Ordered Fuzzy Number (OFN)

The concept of ordered fuzzy numbers, proposed by Prokopowicz, Ślęzak, and Kosiński [84], was introduced with the aim of overcoming the limitations of fuzzy numbers. These include the loss of precision with an increase in the number of operations performed and the inability to solve even linear equations within the set of fuzzy numbers. An ordered fuzzy number (OFN) is an ordered pair $\left(f,g\right)$ of continuous functions f,g:[0,1]→R [84]. The concept has also been described in [85,86].

The set of ordered fuzzy numbers (OFNs) is highly diverse, with only a small proportion of them exhibiting a format that aligns with the conventional fuzzy number format. The set of pairs of continuous functions, where one function is increasing and the other is decreasing, and where the increasing function assumes values that are lower or equal to the decreasing function, forms a subset of the set of OFNs. This subset represents the class of all convex fuzzy numbers with continuous membership functions. These numbers are referred to as proper OFNs.

**Figure 2:**
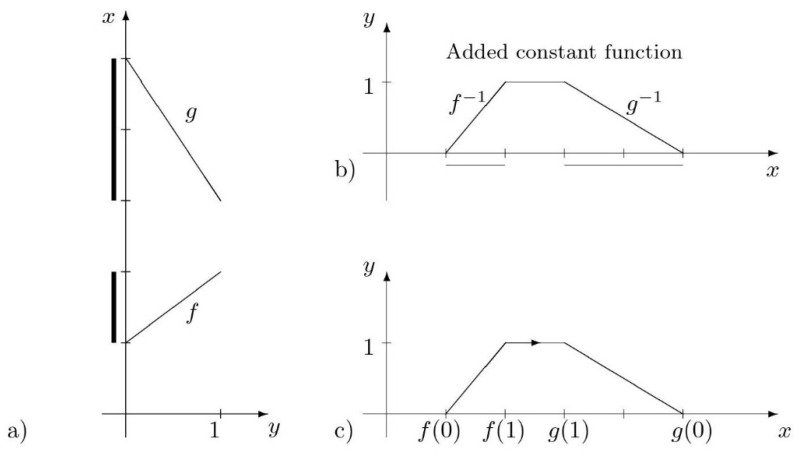
(**a**) Construction of a standard representation of a proper OFN (*f*,*g*), where f is an increasing function, (**b**) OFN presented as classical fuzzy number, (**c**) simplified mark denotes the order of inverted functions [87].

In the case of a proper OFN denoted by A=(f, g) (Figure 2a), a function $\mu_{A}\left(x\right)$ can be defined. The function depicted in Figure 2b is referred to as the standard representation of a proper ordered fuzzy number. It aligns with the concept of a membership function in standard fuzzy numbers. It is notable that the graphical representations of (f, g) and (g, f) are indistinguishable. However, these function pairs define distinct ordered fuzzy numbers due to a property known as direction (also referred to as orientation in some of the literature). This direction is indicated by an arrow in Figure 2c.

A linear proper OFN (f,g) (trapezoidal OFN) is defined as an ordered pair of linear functions, which can be represented by the following vector with four elements of real numbers: $[f\left(0\right),f\left(1\right),g\left(1\right),g\left(0\right)].$ For the sake of simplicity, we shall henceforth denote the trapezoidal OFN as $\left[a,b,c,d\right],$ where a,b,c,d∈R. If a<b≤c<d, the OFN is said to have a positive direction (denoted as A↑). The opposite is also true, as if a>b≥c>d, the OFN is said to have a negative direction (denoted as A↓). If we additionally assume that b=c, we obtain an OFN known as a triangular OFN. This type of number will be used in this paper to represent uncertainty in fuzzy rules that assess the supplier. For the positive direction, a standard representation for triangular  A↑=[a,b,b,c] constitutes the membership function of a standard triangular fuzzy number

(1) $$\mu_{A↑}\left(x\right)=\left\{\begin{array}{ccc} \frac{x-a}{b-a} & for & a<x\leq b\\ \frac{c-x}{c-b} & for & b<x<c\\ 0 & for & x\leq a\;or\;x\geq c \end{array}\right.$$

and for the negative one, a standard representation for triangular A↓=[a,b,b,c] has the following form:

(2) $$\mu_{A↓}\left(x\right)=\left\{\begin{array}{ccc} \frac{x-c}{b-c} & for & c<x\leq b\\ \frac{a-x}{a-b} & for & b<x<a\\ 0 & for & x\leq c\;or\;x\geq a \end{array}\right.$$

The operations of addition, subtraction, and multiplication through a scalar are consistent with the properties of linear operations in four-dimensional vector space.

### 3.2. Ordered Fuzzy Rules

We consider the concept of knowledge representation in the form of conditional ordered fuzzy rules and describe the inference procedure of static fuzzy systems. This implies that the supplier evaluation is solely determined by the current input values representing evaluation values from the perspective of the criteria. The database and the rule-based knowledge representation constitute the knowledge base. In our view, the database contains a set of linguistic values (natural language terms, such as “big and decreasing” and “small and increasing”) of system variables that evaluate suppliers and OFN definitions for these linguistic values. It is proposed that a rule-based knowledge representation be considered for a MISO system, with the following form of ordered fuzzy rule:

(3) $$IF\;\left(x_{1}\;is\;A_{1}\right)\;and\;\ldots \;and\;\left(x_{N}\;is\;A_{N}\right)\;THEN\;y\;is\;B.$$

In this context, the input linguistic variables, represented by the vector $x_{1},\ldots ,x_{N}$, are the criteria used to evaluate suppliers. These criteria may include, for instance, reliability, on-time delivery, and the number of deficiencies. The output linguistic variable y represents the system output. The preliminary evaluations of the supplier, $A_{1},\ldots ,A_{N}\;,\;B$, are the linguistic values identified through triangular OFNs with the membership function (1) or (2) for input criteria and output assessment, respectively. The following is an example of knowledge representation with ordered fuzzy rules regarding supplier evaluation from the perspective of two criteria (quality of services and number of deficiencies):

$$\begin{array}{c} IF\;\left(quality\;of\;services\;is\;“high\;and\;increasing”\right)\;and\;\\ \left(number\;of\;deficiencies\;is\;“low\;and\;decreasing”\right)\\ THEN\;supplier\;assessment\;is\;“\mathrm{high}\;\mathrm{and}\;\mathrm{increasing}”.\\ IF\;\left(quality\;of\;services\;is\;“high\;and\;decreasing”\right)\;and\;\\ \left(number\;of\;deficiencies\;is\;“low\;and\;increasing”\right)\\ THEN\;supplier\;assessment\;is\;“\mathrm{high}\;\mathrm{and}\;\mathrm{decreasing}”. \end{array}$$

The ordered fuzzy model is based on the structure of the Mamdani model, with a different representation of data uncertainty than the classical one. The rule concludes additional information about the positive or negative correlation of the fuzzy numbers. The above example shows that the use of OFNs makes it possible to take into account the direction of change of these fuzzy numbers in the previous time (based on expert opinion or historical data). When analyzing the result of the model in the form of an OFN, we also take into account the dynamics of changes in the value of the fuzzy output. Trends in the value changes of the evaluated criteria affect the supplier’s final score. In addition, a negative direction of change in the final rating may indicate potential problems or higher risks associated with working with a particular supplier.

The knowledge base of ordered fuzzy rules is the basic element needed to implement the fuzzy inference procedure, on the basis of which we obtain a final assessment of a particular supplier.

### 3.3. Inference of Ordered Fuzzy Rules

The fuzzy inference process comprises the following steps, which transform the input data into the corresponding output data (the history of the supplier evaluation values against specific criteria into the final supplier evaluation): fuzzification and evaluation of the strength of the rule mapped to the OFN rule result, fuzzification of the OFN, and aggregation of the results in the rule base. Further details regarding the inference process with ordered fuzzy rules can be found in the authors’ earlier publication [37].

#### 3.3.1. Evaluation of Rule Strength

Consider the following single predecessor of an ordered fuzzy rule: IF (*x* is *A*), where *x* is the input linguistic variable (e.g., quality of services) and *A* is a triangular OFN represented by a linguistic description, e.g., “high and increasing”. Fuzzification represents the initial stage of the fuzzy inference process, wherein a numerical value of the input signal $x^{*}$ (e.g., $x^{*}=8$ with the historical values $\left\{5,7,8\right\}$ of 3 previous observations—a value with a positive trend x↑) is transformed into fuzzy input data. In the case of OFNs, the direction of the OFN is of significant consequence in this process. It is assumed that the expression “x is A” is only meaningful if the numbers $x^{*}$ and *A* have the same direction. In our analysis, the direction of the number $x^{*}$ is calculated from the previous k observations of the value of x. This allows us to represent the trend of past changes in the value of *x*. To illustrate, consider the example of *x*, which represents the time of delivery. The numerical value of $x^{*}$ is 8, and it has a positive direction when the current quality of delivery is assessed at 8 points and an increasing trend in delivery time has been observed in *k* previous observations. The occurrence of a trend is investigated by analyzing the correlation between the input variable x and the time variable t. This is achieved by employing the parametric Pearson correlation coefficient test in the form of the following formula:

(4) $$r_{xt}=\frac{cov(x,t)}{\sigma_{x}\sigma_{t}},$$

where cov(x,t) represents the covariance between the input variables *x* and variable t determining time in the period T=[t−k;t] and $\sigma_{x}{,\sigma }_{t}$ are the standard deviations of *x* and t, respectively. The correlation coefficient is a standardized measure that ranges from −1 to 1. It is assumed that the value of the correlation coefficient affects the direction of change in the following way:

(5) $$\begin{array}{c} direction\;of\;x\;is\;positive\;\left(x↑\right)\;if\;r_{xt}>-0.2\\ direction\;of\;x\;is\;negative\;\left(x↓\right)\;if\;r_{xt}\leq -0.2. \end{array}$$

Returning to the initial example, the input signal $x^{*}=8$ with the historical values in order $\left\{5,7,8\right\}$ of 3 previous observations has a positive trend x↑ because $r_{xt}$ = 0.91.

In the processing of imprecise data, the use of OFNs as an alternative to classical fuzzy numbers is a viable option. However, in instances where the additional information contained in the new model is to be taken into account, direction-sensitive methods are necessary. A method of this nature was proposed by Piotr Prokopowicz in [36]. The primary role is played by a relation called the direction determinant $D_{A}$.

For a triangular OFN A↑=[a,b,b,c], the fuzzification result of $x^{*}$ with a positive direction is a function called a direction determinant $D_{A↑}:(a,b)\to \left(-1,1\right)$, such that

(6) $$D_{A↑}(x^{*})=\left\{\begin{array}{cc} \frac{x^{*}-b}{b-a} & dla\;x^{*}\in (a,b]\\ \frac{x^{*}-b}{c-b} & dla\;x^{*}\in \left(b,c\right) \end{array}\right..$$

In the case of a negative direction of a triangular OFN A↓=[d,e,e,f], we have modified Prokopowicz’s definition of the direction determinant in order to indicate the manner in which a negative trend in a given value affects this value. This influence is assessed by the expert by specifying a value $\rho \in \left(0;0.5\right).$ The larger the value of ρ, the greater the impact of the negative trend on the assessment of that value. Accordingly [37],

(7) $$D_{A↓}(x^{*})=\left\{\begin{array}{cc} \frac{x^{*}-e}{e-f}-\rho & if\;x^{*}\in (f,e]\\ \frac{x^{*}-e}{d-e}-\rho & if\;x^{*}\in \left(e,d\right) \end{array}\right..$$

For the simple rule, the fuzzification result, i.e., the value of the function $D_{A}(x)$ (and thus $D_{A↑}(x)$ or $D_{A↓}(x))$, is also an assessment of the strength of this single ordered fuzzy rule.

Let us consider a complex rule for a MISO-type system in the form (3). Let $(x_{1}^{*}$, …, $x_{n}^{*})$ be a vector of the quantitative system input values—the values of criteria in the supplier assessment. If $\mu_{A_{i}}\left(x_{i}^{*}\right)=0$ for some i=1,…,n, then the given rule is not activated. Otherwise, the strength of this complex ordered fuzzy rule is calculated from the fuzzy AND operator in the predecessor using the following rule:

(8) $$D_{A}=\frac{1}{n}\sum_{i=1}^{n}\varepsilon_{i}D_{A_{i}}\left(x_{i}^{*}\right),$$

where $\varepsilon_{i}=1$ when the *i*-th input is a “benefit” criterion of supplier assessment and $\varepsilon_{i}=-1$ when the *i*-th input is a “cost” one.

#### 3.3.2. Mapped to the Output OFN

The next stage in fuzzy inference involves the implication from the antecedent’s information to the consequent. This step determines OFN to assess the supplier based on the conclusion of an individual rule. This step is called “Directed Inference by the Multiplication with a Shift” (DIMS) by P. Prokopowicz [36]. Let B=[k,l,l,m] be a triangular OFN representing the supplier assessment in the output of rule (3). The resulting OFN $B^{\prime }$ from applying rule (4) cannot be calculated if the rule is not activated. However, if the rule is active, the OFN for the conclusion of rule $B^{\prime }$ can be computed as

(9) $$B^{\prime }=\left\{\begin{array}{cc} {}[k,l,l,m]+\left|D_{A}\right|\left(\left[k,k,k,k\right]-[k,l,l,m]\right) & if\;D_{A}\leq 0\\ {}[k,l,l,m]+\left|D_{A}\right|\left(\left[m,m,m,m\right]-[k,l,l,m]\right) & if\;D_{A}>0 \end{array}\right.$$

for B with a positive direction. To make the implication process more responsive to the direction of OFN, we modified the DIMS procedure for negative-oriented B, as follows:

(10) $$B^{\prime }=\left\{\begin{array}{cc} {}[k,l,l,m]+\left|D_{A}\right|\left(\left[m,m,m,m\right]-[k,l,l,m]\right) & if\;D_{A}\leq 0\\ {}[k,l,l,m]+\left|D_{A}\right|\left(\left[k,k,k,k\right]-[k,l,l,m]\right) & if\;D_{A}>0 \end{array}\right..$$

#### 3.3.3. Defuzzification of Output OFN

The final stage of inference for a single rule is the defuzzification procedure. This is the process of transforming a fuzzy number into a crisp value or number. In the context of supplier evaluation, for example, the defuzzification stage yields an elementary numerical rating for each supplier, obtained as the result of inference from a single rule. We have defined a direction-sensitive defuzzification functional for a linear OFN in the form of $B^{\prime }=[a^{\prime },b^{\prime },b^{\prime },c^{\prime }]$*,* as follows:

(11) $$\varphi \left(B^{\prime }\right)=\frac{\mu a^{\prime }+b^{\prime }+\left(2-\mu \right)c^{\prime }}{3},$$

where μ∈(0,1]. The smaller the μ value, the greater the influence of the direction on the process of defuzzification. In the following empirical example, we utilize the value of μ as 0.8.

#### 3.3.4. Entropy of Results Set

Once the inference has been carried out for each activated ordered fuzzy rule, the Shannon entropy for crisp results can be used to assess the degree of confidence in the received values at the output of the system. The entropy is calculated according to the following formula:

(12) $$H=-\frac{1}{lnN}\sum_{n=1}^{N}\varphi \left({B^{\prime }}_{n}\right)ln(\varphi \left({B^{\prime }}_{n}\right)),$$

where *N* denotes the number of activated rules and $\varphi \left({B^{\prime }}_{n}\right)$ is a numerical rating of the supplier for *n*th elementary rule (n=1,…,N). The calculated entropy falls within the range [0, 1], with a higher value indicating a greater degree of uncertainty in the final supplier evaluation.

#### 3.3.5. Aggregation of Results

In order to obtain the final supplier assessment $y^{*}$, it is necessary to aggregate the values of the numerical rating from the elementary rules in order to produce the result for the entire knowledge base of ordered fuzzy rules. Due to the fact that the values (11) already contain information about the orientation, the aggregation can be performed using a simple arithmetic mean,

(13) $$y^{*}=\frac{\sum_{n=1}^{N}\varphi \left({B^{\prime }}_{n}\right)}{N},\;$$

where N is the number of activated rules, $\varphi \left({B^{\prime }}_{n}\right)$ is a numerical rating of the supplier for the *n*th elementary rule (n=1,…,N), and $y^{*}$ is a final supplier assessment, obtained as a result of the presented inference process for ordered fuzzy rules.

Each activated rule is accompanied by a description of the supplier’s evaluation, presented in the form of an OFN in the output. As we know, each number of OFNs has a direction (positive or negative). The prevalence of directions can provide insight into the general trend of changes in evaluation. A preponderance of positive directions is indicative of a favorable phenomenon, while a preponderance of negative directions can serve as an early warning indicator, indicating emerging issues before they become significant supply threats.

## 4. Empirical Example of Implementation

The following section is intended to illustrate an example of the application of the proposed supplier evaluation system and display problematic situations in which the system has advantages over existing solutions. For the purposes of this discussion, we shall assume that we are dealing with an electronic equipment manufacturing company that works with a number of suppliers of electronic components. In order to facilitate the process of selecting and evaluating suppliers, the company uses an evaluation system based on three criteria.

**Local hiring**: Percentage of employees residing in the region where the company operates (e.g., 95% of employees are from the local area). This social criterion is designed to contribute to the development of the local community. Furthermore, it reduces the environmental impact associated with long commutes.

**Completeness**: Percentage of deliveries containing all ordered items (e.g., 98% of deliveries contain a full order).

**Defects**: Percentage of defective electronic components in shipments (e.g., 2% of processors are defective).

The aforementioned criteria are the input linguistic variables for the proposed supplier evaluation system, which are described by three descriptive values (low, medium, high), identified with triangular OFNs. The database associated with these variables is described in Table 2, Table 3 and Table 4. It can be noted that the presentation of criteria assessments in the form of OFNs also allows for the inclusion of trends in the changes of these assessments on the basis of historical data. The parameters of the triangular fuzzy numbers used slightly exceed the specified range due to the avoidance of the divide-by-zero error in Formulas (6) and (7).

The concept is based on the assumption that the output of the system is a linguistic variable that evaluates the supplier in a holistic manner. This variable is defined by OFN and, as a result, it also has an orientation that denotes the overall direction of change in the evaluation. The database associated with this variable is described in Table 5. Once more, the parameters of triangular ordered fuzzy numbers exceed the 0–100% scale due to the defuzzification method (see (11)), which averages the range of output values obtained.

**Figure 3:**
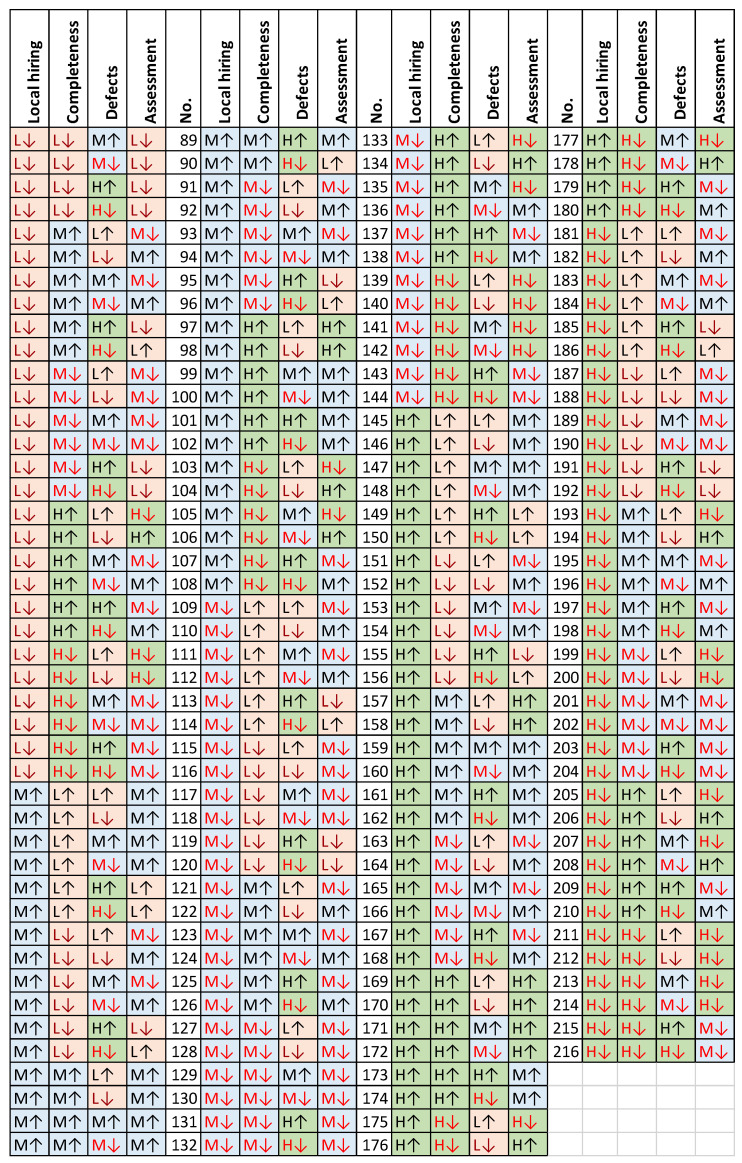
Knowledge base for supplier assessment system.

In examining the aforementioned database, the knowledge base for such assumptions was established through an independent process by three experts. In the majority of cases, a consensus was reached regarding the rules, but in instances of divergence, a definitive conclusion was determined through joint discussion. This approach resulted in the establishment of a coherent knowledge base comprising 216 elementary rules (Figure 3).

To illustrate, the 24th elementary rule (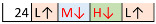) can be interpreted as follows: when local hiring is rated as “low and increasing”, completeness is rated as “medium and decreasing”, and defect is rated as “high and also decreasing”, then the supplier evaluation is rated as “low and increasing”. The combination of low local hiring, high defect rates, and medium completeness results in an overall low rating for the supplier. Conversely, an upward trajectory in the local hiring rating and a reduction in the number of defects, which are two key criteria from the perspective of experts, contribute to a positive orientation in the final rating.

In order to carry out the inference process, the following assumptions regarding the impact factor and the constant *ε* are made (Table 2, Table 3 and Table 4). The impact factor values suggest that the negative direction of the completeness criterion (*ρ* = 0.3) has the greatest impact on the assessment, while the negative direction of the local hiring criterion has the least impact (*ρ* = 0.2). However, these are not really significant differences. The constant ε=−1 for the defects criterion ensures that it is a cost criterion—the higher the value of defects, the weaker the supplier’s assessment. The other criteria are profit criteria (ε=1), and, therefore, the larger the values, the better the supplier’s assessment.

**Figure 4:**
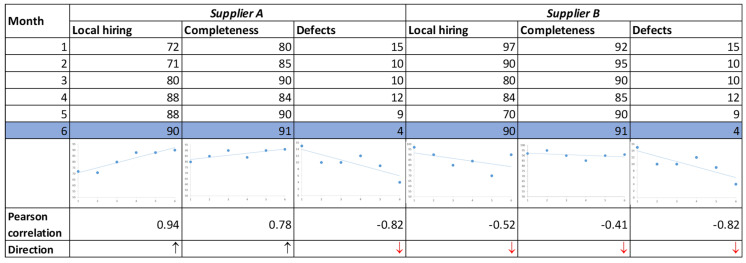
Data on cooperation with suppliers A and B.

Consider the example of evaluating two suppliers, A and B. We evaluate the suppliers in the sixth month of cooperation. We determine the trend of evaluations based on the six-month results (k=6). Figure 4 shows data on cooperation with suppliers A and B over the past six months.

The evaluation of suppliers based on classical evaluation methods (e.g., MCDM methods, fuzzy logic systems with classical fuzzy sets, neural network systems) is limited to the assessment of static data (current data describing the given criteria). Consequently, suppliers A and B are evaluated in a similar manner, given that their data in the sixth month are identical.

The proposed system allows for the differentiation between supplier A and supplier B, taking into account the historical changes in supplier evaluation. It is notable that the months preceding the evaluation indicate a favorable direction of change in the criterion values for supplier A, with positive trends for profit-type criteria (local hiring, completeness) and negative trends for the cost-type criterion (defects). With regard to supplier B, the initial two criteria are negatively oriented, which is a cause for concern. Despite the satisfactory current ratings, the negative trajectory of past ratings may result in difficulties in maintaining effective collaboration with this supplier in the future.

The numerical data from Figure 4, both historical and current, are used to evaluate both suppliers, with eight rules being activated for each (Table 6). Nevertheless, a distinct set of rules is triggered for each supplier. In accordance with the inference process, the OFN output is mapped in each rule, which, following defuzzification, indicates the definitive assessment of the supplier for elementary rules (Table 6). Ultimately, the application of formula (13) yields the following supplier evaluations: supplier A has 68.28 points and supplier B has 65.02 points. The evaluation results for both suppliers differ slightly (by 3.26 points) in favor of supplier A. This is due to the influence of the trend of changes in values based on the six-month period. Furthermore, the prevalence of the negative trend (100% negative) for supplier B serves as an indicator for the company to initiate the identification of issues related to the supplier’s performance.

The entropy-type fuzziness index indicates that the degree of uncertainty associated with the assessment of supplier A is less than that of supplier B. It can be posited that higher entropy is indicative of greater uncertainty regarding the final supplier assessment. The degree of uncertainty is measured here by the degree of crisp fuzziness of the results obtained through inference for individual ordered fuzzy rules.

Finally, the supplier evaluation can be conventionally divided into three groups:

- 0.00–33.33 points: red group (immediate supplier rejection);
- 33.34–66.66 points: yellow group (potential problems);
- 66.67–100.00 points: green group (reliable supplier).

In the example above, supplier A was identified as a reliable supplier and thus assigned to the green zone, whereas Supplier B was placed in the yellow zone, indicating the presence of potential issues. In the case of a classical fuzzy inference system, it is not possible to distinguish between the two suppliers, and thus they both receive the same evaluation. It can be observed that the proposed method of supplier evaluation has the potential to offer an advantage in this regard. The incorporation of OFNs within a fuzzy inference system enables the consideration of the trajectory of alterations in the values of individual evaluation criteria, which subsequently influences the differentiation of the final supplier evaluations. The resulting measure is founded upon a more substantial corpus of information, which in turn has a favorable impact on its reliability.

## 5. Discussion

The evaluation of suppliers represents a fundamental instrument for businesses seeking to make well-informed decisions regarding their supply chains. By understanding the strengths and limitations of different methods, companies can adapt their approach to align with their specific needs and priorities. A well-designed evaluation process can significantly enhance the selection of suppliers, leading to increased efficiency, cost savings, and overall business success. Supplier evaluation is a multifaceted process that is pivotal to effective supply chain management. The literature reveals a variety of methods and criteria used to assess suppliers, underscoring the importance of a comprehensive and dynamic approach.

This paper presents an integrated approach to the evaluation of suppliers within the context of the supply chain. The specific criteria system and alternative suppliers have been defined for the purpose of evaluating the aforementioned problem within the context of the supply chain. The parameters of the criteria were defined using ordered fuzzy numbers, thus addressing the issue of expressing uncertain decision making information. This offers a means of representing subjective preferences. This approach can be regarded as a dynamic methodology for supplier evaluation within the context of the supply chain, which represents a significant advantage. The knowledge base for the supplier evaluation system was created on the basis of expert opinion using ordered fuzzy numbers (OFNs). The incorporation of OFNs into a fuzzy model enables a reduction in the complexity of the knowledge base in comparison to a classical fuzzy system. This makes the model more accessible to users, as it requires only basic arithmetic operations in the inference process. The application of the proposed approach was demonstrated using a numerical example to determine the most suitable supplier in the supply chain.

## 6. Conclusions

This paper presents a comprehensive overview of the various methods employed in the evaluation of suppliers. The research presents a novel approach to supplier evaluation and selection, which employs an ordered fuzzy decision making system. This approach addresses several key challenges in the field of supplier assessment, including the following:

- The utilization of a fuzzy system with ordered fuzzy numbers (OFNs) in the context of supplier assessment represents an innovative application of the concept, contributing to the advancement of fuzzy logic techniques in supplier evaluation.
- The presented inference process based on the created ordered fuzzy rules takes into account the dynamic changes in the value of assessment parameters in the overall supplier assessment, allowing for the differentiation of suppliers based on current and historical data. This approach to supplier assessment can assist organizations in mitigating the risks associated with supplier selection and enhancing their overall supply chain performance.
- The proposed system effectively incorporates multiple criteria for supplier evaluation, including traditional factors, such as completeness and delivery defects, and social factors, such as local hiring. This criteria can be also easy expanded to include new factors, such as flexibility and sustainability. This allows for more effective management of uncertainty and subjectivity in the decision making process.
- The application of supplementary techniques, such as entropy and trends of changes in the assessment, enables a more precise delineation of the uncertainty associated with the assessment and its underlying trends. This additional information has a beneficial impact on the decision making process.

In conclusion, this research makes a significant contribution to the field of supplier evaluation by introducing a robust, flexible, and comprehensive decision making system.

The utilization of ordered fuzzy numbers and the incorporation of sustainability factors make this approach particularly relevant in the context of modern business environments. The proposed approach has the potential to enhance the reliability of appropriate supplier evaluation outcomes.

Nevertheless, this approach is not without its inherent constraints. Firstly, the evaluation criteria proposed in this paper consider only three dimensions, which may result in an incomplete assessment of alternative suppliers. Secondly, the evaluation criteria are assigned identical weights, which fails to account for the influence of experts on the weighing procedure. In order to create a tool for more accurate supplier evaluation, we plan to increase the number of criteria considered, apply an integrated method for calculating criteria weights, and examine specific industries in more detail. Furthermore, we intend to consider incorporating emerging trends, such as the integration of artificial intelligence and big data, into supplier evaluation processes.

## Figures and Tables

**Table 1:** The key analytical approaches and methods used for supplier selection (ref. [3,5,6]).

Analytical Approach	Method	Description
Multi-Criteria Decision Making (MCDM)	Analytic Hierarchy Process (AHP)	A structured technique for organizing and analyzing complex decisions based on pairwise comparisons and hierarchical structuring of criteria.
	Analytic Network Process (ANP)	An extension of AHP that considers interdependencies among criteria and alternatives.
	TOPSIS	Technique for Order of Preference by Similarity to Ideal Solution—ranks alternatives based on their distance from the ideal solution.
	DEMATEL	Decision Making Trial and Evaluation Laboratory—used to analyze and visualize the structure of complex causal relationships among criteria.
Mathematical Programming	Linear Programming (LP)	A mathematical approach to optimizing a linear objective function subject to linear equality and inequality constraints.
	Integer Programming	Similar to LP but involves decision variables that are integers.
	Goal Programming	Extends LP by incorporating multiple objectives with priority levels.
Data Envelopment Analysis (DEA)	Basic DEA	A non-parametric method to evaluate the relative efficiency of suppliers by comparing multiple input–output measures.
	Network DEA	An extension of DEA that considers the internal structure and interdependencies within a network of decision making units.
Artificial Intelligence (AI) Methods	Neural Networks	Machine learning models that simulate the decision making process by identifying patterns and learning from data.
	Expert Systems	Rule-based systems that use knowledge bases to emulate the decision making ability of human experts.
Hybrid Models	AHP-DEA	Combines AHP for weighting criteria and DEA for evaluating efficiency, leveraging the strengths of both methods.
Discrete Choice Analysis (DCA)	DCA Models	Econometric methods used to model decision making where choices are discrete and based on the attributes of suppliers and the decision context.
Sustainability- and Risk-Focused Approaches	Green Supplier Selection Models	Models that incorporate environmental and sustainability criteria in addition to traditional supplier selection factors.
	FMEA-Based Models	Use Failure Mode and Effect Analysis (FMEA) to evaluate and rank suppliers based on the likelihood and impact of potential risks.

**Table 2:** Database for criterion local hiring (“benefit” criterion).

Local Hiring	Range	[50%, 100%]
	Impact Factor *ρ*	0.2
	Constant *ε*	1
Description	Direction	Parameters
Low and increasing {L↑}	Positive	[49.9, 50, 50, 75]
Medium and increasing {M↑}	Positive	[50, 75, 75, 100]
High and increasing {H↑}	Positive	[75, 100, 100, 100.1]
Low and decreasing {L↓}	Negative	[75, 50, 50, 49.9]
Medium and decreasing {M↓}	Negative	[100, 75, 75, 50]
High and decreasing {H↓}	Negative	[100.1, 100, 100, 75]

**Table 3:** Database for criterion completeness (“benefit” criterion).

Completeness	Range	[70%, 100%]
	Impact Factor *ρ*	0.3
	Constant *ε*	1
Description	Direction	Parameters
Low and increasing {L↑}	Positive	[69.9, 70, 70, 90]
Medium and increasing {M↑}	Positive	[70, 90, 90, 100]
High and increasing {H↑}	Positive	[90, 100, 100, 100.1]
Low and decreasing {L↓}	Negative	[90, 70, 70, 69.9]
Medium and decreasing {M↓}	Negative	[100, 90, 90, 70]
High and decreasing {H↓}	Negative	[100.1, 100, 100, 90]

**Table 4:** Database for criterion defects (“cost” criterion).

Defects	Range	[0%, 30%]
	Impact Factor *ρ*	0.25
	Constant *ε*	−1
Description	Direction	Parameters
Low and increasing {L↑}	Positive	[−0.1, 0, 0, 5]
Medium and increasing {M↑}	Positive	[0, 10, 10, 20]
High and increasing {H↑}	Positive	[20, 30, 30, 30.1]
Low and decreasing {L↓}	Negative	[5, 0, 0, -0.1]
Medium and decreasing {M↓}	Negative	[20, 10, 10, 0]
High and decreasing {H↓}	Negative	[30.1, 30, 30, 20]

**Table 5:** Database for supplier assessment.

Assessment (Range [0, 100])	Direction	Parameters
Low and increasing {L↑}	Positive	[−25, 0, 0, 50]
Medium and increasing {M↑}	Positive	[25, 50, 50, 75]
High and increasing {H↑}	Positive	[50, 100, 100, 125]
Low and decreasing {L↓}	Negative	[50, 0, 0, −25]
Medium and decreasing {M↓}	Negative	[75, 50, 50, 25]
High and decreasing {H↓}	Negative	[125, 100, 100, 50]

**Table 6:** The result of the inference process.

Supplier A						Supplier B					
No. of Activated Rules	Local Hiring	Completeness	Defects	Assessment	Crisp Assessment for Elementary Rule	No. of Activated Rules	Local Hiring	Completeness	Defects	Assessment	Crisp Assessment for Elementary Rule
86	M↑	M↑	L↓	M↑	52.8333	128	M↓	M↓	L↓	M↓	45.6111
88	M↑	M↑	M↓	M↑	63.7222	130	M↓	M↓	M↓	M↓	57.6667
98	M↑	H↑	L↓	H↑	81.6528	140	M↓	H↓	L↓	H↓	71.5417
100	M↑	H↑	M↓	M↑	55.9444	142	M↓	H↓	M↓	H↓	89.7639
158	H↑	M↑	L↓	H↑	81.6528	200	H↓	M↓	L↓	H↓	71.5417
160	H↑	M↑	M↓	M↑	55.9444	202	H↓	M↓	M↓	M↓	48.7778
170	H↑	H↑	L↓	H↑	66.9306	212	H↓	H↓	L↓	H↓	58.4861
172	H↑	H↑	M↓	H↑	87.5417	214	H↓	H↓	M↓	H↓	76.7639
Final assessment:					68.2778	Final assessment:					65.0191
Trends of changes in assessment:					100% positive	Trends of changes in assessment:					100% negative
Entropy (uncertainty of final assessment):					0.8333	Entropy (uncertainty of final assessment):					0.9167

## Data Availability

The data presented in this study are available on request from the corresponding author.
